# Likelihood based inferences for trials incorporating participant’s treatment choice

**DOI:** 10.1016/j.conctc.2024.101306

**Published:** 2024-05-15

**Authors:** Rouba Chahine, Inmaculada Aban

**Affiliations:** a Center of Clinical Research, RTI International, Research Triangle Park, NC, USA; b Department of Biostatistics, University of Alabama at Birmingham, Birmingham, AL, USA

**Keywords:** Covariates, Doubly randomized, Exponential family, Likelihood ratio test, Two-stage random choice trials

## Abstract

Randomized clinical trials are the gold standard for clinical trials as they reduce bias and minimize variability between different arms of a study. One of the drawbacks of these designs is their lack of flexibility to incorporate participant’s treatment choice, which may reduce recruitment rates and/or reduce participant’s tolerance if they receive a non-preferred treatment. Designs incorporating choice allow a subset of participants to choose their preferred treatment. Most of the current methods to analyze these types of designs are based on an ANOVA approach that do not allow for inclusion of covariates in the model. In this paper, we propose an alternative approach based on likelihood methods that can be used with a broad class of distributions and allow for inclusion of covariates and multiple study arms in the model. Using simulations, we evaluate these methods for a variety of continuous and categorical outcomes. Finally, we illustrate these methods by analyzing change in six minute walking distance from a behavioral intervention study for women with heart disease.

## 1 Introduction

A standard component of informed consent in randomized clinical trials (RCT) is participant’s awareness of all procedures or treatments that might benefit them. This knowledge may decrease compliance or motivation to continue study participation when randomized to non-preferred treatment. This is of particular concern in unblinded studies. Designs incorporating patient’s treatment choice are gaining popularity in part due to efforts of organizations like the Patient-Centered Outcomes Research Institute whose mission is to “*help people make informed healthcare decisions, and improves healthcare delivery and outcomes*” [1]. Since the 1970s, researchers have been speculating about the effect of patient’s treatment choice on the outcomes of clinical trials, see for instance [2], [3], [4], and [5].

Rücker’s design gained popularity over the last decade, prompting statisticians to investigate methods to analyze this kind of design. Her two-stage RCT design incorporates participant’s treatment choice in a study. In this type of design, participants are randomized to either a random group or a choice group. Next, participants in the random group are randomized to one of two treatments following usual RCT process, while participants in the choice group are asked to choose their treatment.

The advantage of such design is that it allows the investigator an opportunity to assess the effect of choice on the study outcome. Consequently, the main focus shifts away from testing for treatment effect to testing for preference effect, defined as the interaction between treatment and choice. Otherwise, investigators aiming to test for treatment effect only can combine both groups and ignore participant’s choice.

Rücker used analysis of variance (ANOVA) approach to study three effects in two-stage RCT: treatment, selection (choice), and preference. Under the ANOVA assumptions, the outcomes are assumed to be normally distributed, and the variances are equal in all subgroups. Additionally, the ANOVA approach assumes that the sum of all the treatments equal to zero and if patients in the random group are given the choice, the proportion of participants in the random group choosing one treatment over another will be the same as the choice group. Later, Cameron et al. [2] extended Rücker’s work to binary outcomes and allowed for stratification in the model. Shi et al. [6] extended it to count outcomes, more specifically Poisson. To date, we do not believe anyone extended the ANOVA method for these designs to a version that accommodates covariates.

An alternative to the ANOVA approach is based on likelihood function. Long et al. [7] proposed a likelihood model for assessing the preference effect on the outcomes of interest in two-stage RCT. They modified some of Rücker’s assumptions allowing for covariates to be associated with treatment selection. However, this flexibility introduced the issue of missingness because patient’s choice is not usually recorded for participants in the random group. The authors solved this problem by using an expectation–maximization (EM) algorithm in which the E-step replaces missing values by their conditional expectations given the observed data and the M-step maximizes the expected log likelihood to estimate the model parameters. One of the concerns of the EM algorithm as proposed by Long et al. is that the imputation of missing participant’s choice variable for the random group which depends on assumptions may not be valid in some cases. In addition, it is computationally intensive and requires creating a program for implementation of this algorithm. In our review of literature, we unfortunately have not found a software or program available for public use. Other methods used by researchers to analyze data from designs that incorporates patient’s treatment choice are based on standard regression models adjusting for participant’s group randomization and for the treatment received (for instance see [8], [9]).

In this paper, our goal is to propose a likelihood-based approach to analyze hybrid designs that incorporate participant’s treatment choice. We focus on likelihood-based procedure to test for preference effect, i.e., if patient’s choice has a modifying effect on the treatment effect. Our proposed likelihood method assumes no information about the participant’s choice of treatment for the random group and hence avoiding the need for imputation. It allows for the inclusion of multiple arms in the study as well as for the inclusion of covariates associated with the outcome of interest and interaction terms. We note here that our method does not allow for the estimation of the selection (choice) effect because we do not use a choice parameter in our methods. Methods by Rücker and Long included the choice parameters by replacing missing values based on specific assumptions, Rücker by using the principle of randomization and Long by using EM algorithm. Our methods do not make any assumptions other than large sample and distribution of the data to estimate the needed effects giving it a wider applicability while still allowing it to estimate the treatment and preference effect.

In this paper, we start by writing a conditional likelihood for any outcome with distribution that follows regularity conditions. We estimate the model parameters associated with the treatment and preference effects using maximum likelihood methods. In Section 3, we use simulations to evaluate the suggested methods for Normal, Bernoulli, and Poisson outcomes, and to evaluate type I error and power. In Section 4, we apply the proposed methods to Women Take PRIDE data [10].

## 2 Model assumptions & methods

### 2.1 Assumptions and the conditional likelihood

For convenience, we assume two treatments. We denote random variables and their observed values with upper and lower case letters, respectively. Let T and C be two indicator variables for treatment received and treatment selected. Specifically, $t_{i}=1$ if the ith participant receives treatment A and zero otherwise (treatment B), and $c_{i}=1$ if the participant selects treatment A and zero otherwise. R is a group randomization indicator for the arm of the study the participant was randomized into ($r_{i}=1$ for the random arm and $r_{i}=0$ for the choice arm). N is the study sample size, m=θN randomized to the choice group and n=(1−θ)N to the random group, with θ defined as the proportion of participants randomized to the choice group. For j=1,2, $m_{j}$ is the number of participants in each of the choice arms and $n_{j}$ is the number of participants in each of the random arms. ϕ is the proportion of participants in the choice arm who chose treatment A so that $m_{1}=\phi \theta N\;\&\;m_{2}=\left(1-\phi \right)\theta N$. No restriction is imposed on ϕ, and its value will be estimated from the observational data

In our work, we are making the following assumptions. All participants in the choice arm will make a choice and will receive the treatment of their choice. Participants in the random arm are not asked about their choice. Let Y represents the outcome of interest, and X vector of covariates. The conditional expected value of Y for the choice and random groups are respectively given by,

(1) $$\mu_{C}=E\left(Y_{i}|T_{i},X_{i},R_{i}=0\right)$$

(2) $$\mu_{R}=E\left(Y_{i}|T_{i},X_{i},R_{i}=1\right)$$

with

(3) $$g\left(\mu_{C}\right)=\beta_{C0}+\beta_{CT}T_{i}+\alpha_{C}X_{i}$$

(4) $$g\left(\mu_{R}\right)=\beta_{R0}+\beta_{RT}T_{i}+\alpha_{R}X_{i}$$

where g(.) is a monotonic link function, $\beta_{C0}$ and $\beta_{R0}$ are functions (g) of the means for participants on treatment B (i.e., $t_{i}=0$) in choice and random group, respectively, controlling for covariates, and $\beta_{CT}$ and $\beta_{RT}$ are functions of the coefficients representing the treatment effects (i.e., $t_{i}=1$). $\alpha_{R}$ and $\alpha_{C}$ are vectors of added effects of the covariates in the model. Eqs. (3), (4) can be extended to include interaction between the covariates and treatment.

Let $f_{C}$ and $f_{R}$ represent the distribution of the outcome for the choice and random groups, respectively. We assume that these distributions each follow regularity conditions (see [11], page 516 for more details). Of particular interest are distributions belonging to the exponential family. Let ($\mathrm{\beta ,\alpha})=\left(\beta_{C},\beta_{R},\alpha_{C},\alpha_{R}\right)=$ $\left(\beta_{C0},\beta_{CT},\beta_{R0},\beta_{RT},\alpha_{C},\alpha_{R}\right)$. We write the conditional likelihood as,

$$\begin{array}{c} f\left(Y|T,R,\beta ,\alpha \right)=\\ \overset{N}{\underset{i=1}{\prod }}{\left(f_{C}\left(y_{i}|t_{i},\beta_{C},\alpha_{C}\right)\right)}^{\left(1-r_{i}\right)}{\left(f_{R}\left(y_{i}|t_{i},\beta_{R},\alpha_{R}\right)\right)}^{r_{i}} \end{array}$$

If both $f_{C}$ and $f_{R}$ are members of the exponential family, then their joint distribution is also a member of the exponential family. Next, we write the log-likelihood as,

(5) $$\begin{array}{c} \ell =\log L\left(\beta ,\alpha |y_{i},t_{i}\right)=\\ \overset{N}{\underset{i=1}{\sum }}\left[\left(1-r_{i}\right)\log\left(f_{C}\left(y_{i}|t_{i},\beta_{C},\alpha_{C}\right)\right)+r_{i}\log\left(f_{R}\left(y_{i}|t_{i},\beta_{R},\alpha_{R}\right)\right)\right] \end{array}$$

To estimate parameters in the model, we use the maximum likelihood estimator (MLE). Due to the lack of closed formula for the MLE, we use numerical methods to obtain the estimates. We use the R function [12], using a quasi-Newton algorithm, bounded Broyden–Fletcher–Goldfarb–Shanno (L-BFGS-B).

### 2.2 Likelihood Ratio Test (LRT)

Using the proposed method, one may test any effect of interest represented in the model. Following LRT method, we have the test statistic,

$$\lambda \left(y\right)=\frac{L\left({\overset{ˆ}{\beta }}_{H_{0}},{\overset{ˆ}{\alpha }}_{H_{0}}|y,t,r\right)}{L\left(\overset{ˆ}{\beta },\overset{ˆ}{\alpha }|y,t,r\right)},$$

where $H_{0}$ represents the null hypothesis.

Under the regularity conditions, −2log(λ(y)) converges to a chi-square distribution with d degree of freedom [11], where d calculated as the difference between the number of free parameters specified under the unrestricted model and those under the null hypothesis. The null hypothesis will be rejected at α level when

(6) $$-2\log\left(\lambda \left(y\right)\right)>\chi_{d,\alpha }^{2}$$

**Fig. 1:**
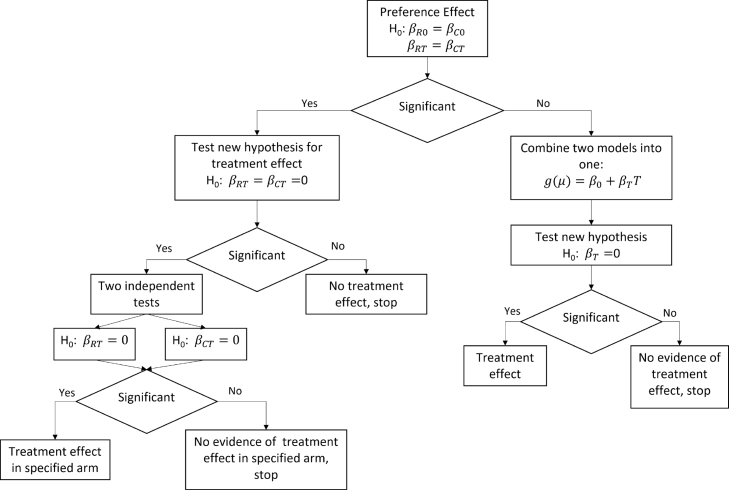
Flow chart illustrating testing steps for the preference effect.

One of the main aims of conducting trials incorporating participant’s choice is to study the effect of choice on treatment. To perform such a test, we write the hypothesis of no preference effect (i.e, test if the two treatment A and B are the same in both groups choice and random) as $H_{0}:\beta_{R0}=\beta_{C0}$ and $\beta_{RT}=\beta_{CT}$ versus the alternative that at least one equality fails. In Fig. 1, we provide a flow chart detailing suggested steps/tests to be performed. As this figure illustrates, we propose different approaches for testing of treatment effect based on the significance of the test of preference effect. To account for multiple testing of nested hypotheses of the left side of the flow chart, we propose not performing any adjustment for the primary hypothesis of preference [13], however for all subsequent secondary analyses nested below the preference effect, we propose adjusting alpha level using approach similar to Holm’s [14]. The stepwise adjustment should be conducted in the order of nesting, concluding the testing process when a non-significant result is obtained. It is assumed that all subsequent tests are not significant after reaching this point.

### 2.3 Special cases

For this paper, we are interested in illustrating the proposed method to commonly used distributions to model outcomes such as Normal, Bernoulli, and Poisson. These distributions are members of the exponential family and satisfy the regularity conditions. In the proposed LRT method, each of the mentioned distributions requires different link functions (g() function in Eqs. (3), (4)). In Table 1, we provide the probability density functions and their associated link functions.

**Table 1:** Distributions and their associated link functions for the ith participant in the κ group (κ=C,R).

Distribution	Probability function	Link function
Normal^a^	$f_{\kappa }\left(y_{i}\right)=\frac{1}{\sqrt{2\pi \sigma^{2}}}e^{-{\left(y_{i}-\mu_{\kappa }\right)}^{2}/\left(2\sigma^{2}\right)}$	Identity, $\mu_{\kappa }=\beta_{\kappa 0}+\beta_{\kappa T}t_{i}$
Bernoulli^b^	$f_{\kappa }\left(y_{i}\right)=\mu_{\kappa }^{y_{i}}{\left(1-\mu_{\kappa }\right)}^{\left(1-y_{i}\right)}$	Logit, $\log\left(\frac{\mu_{\kappa }}{1-\mu_{\kappa }}\right)=\beta_{\kappa 0}+\beta_{\kappa T}t_{i}$
Poisson^c^	$f_{\kappa }\left(y_{i}\right)=\frac{e^{-\mu_{\kappa }}\mu_{\kappa }^{y_{i}}}{y_{i}!}$	Log, $\log\left(\mu_{\kappa }\right)=\beta_{\kappa 0}+\beta_{\kappa T}t_{i}$

a $-\infty <y_{i}<\infty \text{;}\;-\infty <\mu_{\kappa }<\infty$.

b $y_{i}=0,1\text{;}\;0<\mu_{\kappa }<1$.

c $y_{i}=0,1,2,\ldots \text{;}\;0<\mu_{\kappa }<\infty$.

## 3 Simulation studies

In this section, we start by examining the estimates of the MLEs using Quasi-Newton algorithm with L-BFGS-B. Next, we investigate the properties of LRT with regard to type I and power using continuous outcomes (Normal) and discrete outcomes (Bernoulli & Poisson). To determine the number of repetitions needed for the simulations, we relied on formulae provided by Morris et al. [15]. The recommended number of repetitions for type I and power analysis is 3600 repetitions for 90% power size. In this paper, we decided to run 4000.

### 3.1 Evaluating the LRT

The proposed LRT method can be used to test different effects of interest: treatment effect, preference effect, added effect of covariates on treatment effect, etc. In this section, we focus on the preference effect. We first look at the estimates of the parameters for models with and without covariates. Next, we assess type I error and power for the preference effect without covariates which will allow us to compare the results between the LRT and ANOVA approaches. In other words, we test the null hypothesis $H_{0}:\beta_{R0}=\beta_{C0}$ and $\beta_{R0}=\beta_{CT}$ versus the alternative that at least one equality fails. In all simulations, each single experiment consists of 4000 simulated trials with N patients. The percent of participants randomized to random and choice group, θ, is set to 50%. Sample size for participants on treatment A in the random group is set to $n_{1}=n/2$, while number of participants on treatment A in the choice group is randomly generated from a Binomial distribution with m=N/2 and varying choice proportion ϕ. For type I error and power, we run each simulation 10 times using different seeds to account for simulation variations and compare the LRT results with results based on ANOVA methods.

#### 3.1.1 Simulated properties of estimators

To examine the estimates, we ran simulations using different distributions. In this paper, we present the results of Bernoulli with covariates using different proportions for the different arms of the study (0.27 and 0.33) considering different type of covariates (continuous and categorical). In the supporting materials (see Appendix A), we present the results of additional simulations using different distributions. For all the simulations, each data set used consisted of 400 observations. Sample size for the choice group was randomly generated from binomial with n=200 and proportion of participants choosing one treatment over another to 40%. Table 2 summarizes the average, standard deviation, and percentiles (2.5th and 75th) of the simulated distribution of the parameter estimates with the true value of the parameters. Overall, the performance of the estimators seems satisfactory.

**Table 2:** Summary of the simulated distribution of the MLEs based on 4000 replications of Bernoulli data with covariates.

	Case 1: Continuous covariate			
	True value	Estimates (Std)	2.5 Percentile	97.5 Percentile
BetaR0	−1	−1.010 (0.366)	−1.743	−0.309
BetaRT	0	0.001 (0.3)	−0.582	0.576
BetaC0	−1	−1.010 (0.354)	−1.711	−0.330
BetaCT	0.3	0.311 (0.305)	−0.290	0.911
Alpha	0.01	0.010 (0.007)	−0.004	0.025

	Case 2: Binary covariate			
	True value	Estimates (Std)	2.5 Percentile	97.5 Percentile

BetaR0	−1	−1.015 (0.255)	−1.528	−0.542
BetaRT	0	−0.002 (0.321)	−0.649	0.633
BetaC0	−1	−1.014 (0.241)	−1.510	−0.553
BetaCT	0.3	0.297 (0.317)	−0.319	0.932
Alpha	0.1	0.101 (0.233)	−0.326	0.547

#### 3.1.2 Type I error

Empirical type I errors were computed as the proportion of rejection using the cutoff based on a chi-squared distribution with an area of 0.05 to the right and two degrees of freedom (Eq. (6)). ϕ was varied between 10% to 90% in increments of 10%. Sample sizes considered were 100, 200, and 400. For the Normal outcomes, independent observations were simulated using parameters $\left(\beta_{C0},\beta_{CT},\beta_{R0},\beta_{RT}\right)$ = (12, 0, 12, 0) and standard deviation of 5. To produce comparable simulations in the case of Poisson, we set the parameters $\beta_{C0}=\beta_{R0}=2.5$ considering the log link function (log(12)≈2.5). For the case of Bernoulli, we simulated data with proportion greater than 0.5 to deviate from a symmetrical distribution. For a proportion close to 0.6, we set the parameters to (0.3, 0, 0.3, 0) considering the logit link function (Table 1). Simulated results using LRT were compared to simulated results using the ANOVA approach.

**Fig. 2:**
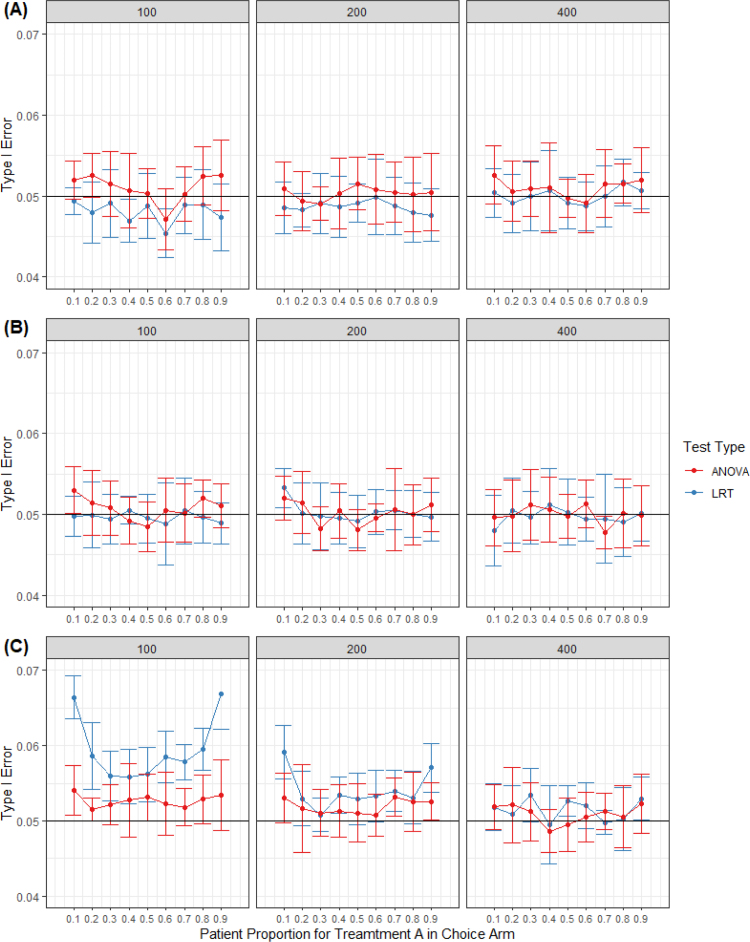
Boxplots for simulated type I error at 0.05 level for preference effect for varying sample sizes. A, Normal ; B, Poisson; C, Bernoulli.

For the Normal and Poisson distributed data, ANOVA and LRT approaches performed about the same with respect to simulated type I error rates, with the LRT performing slightly better for extreme cases of ϕ (Fig. 2). In the case of Bernoulli, both LRT and ANOVA are inflated for smaller sample of 100, with LRT being more inflated. For the larger samples of 200 and 400, this issue dissipates and both methods perform similarly. We note that when ϕ=10% and N=100, we only expect five participants to be on treatment A in the choice group, this sample is too small for the large sample approximation of the LRT to perform well. In this case, sample size needs to be increased significantly to use the LRT method.

#### 3.1.3 Power

We investigated the simulated power for the preference effect using the proposed LRT method, and compared it to power simulated using the ANOVA approach and power based on the standard sample size formula in Eq. (7) (see [16] for details).

(7) $$\begin{array}{c} N_{\pi }=\frac{{\left(Z_{1-\alpha /2}+Z_{1-\beta }\right)}^{2}}{4{\left(\Delta \pi \right)}^{2}\theta \phi^{2}{\left(1-\phi \right)}^{2}}[\phi \sigma_{11}^{2}+\left(1-\phi \right)\sigma_{22}^{2}\\ +2\frac{\theta }{\left(1-\theta \right)}\left(\phi^{2}\sigma_{1}^{2}+{\left(1-\phi \right)}^{2}\sigma_{2}^{2}\right)+\frac{{\left(\phi^{2}d_{1}-{\left(1-\phi \right)}^{2}d_{2}\right)}^{2}}{\phi \left(1-\phi \right)}], \end{array}$$

where Δπ is preference effect, σ standard deviations for the four arms of the study with double indices used for the choice group and a single index for the random group, $d_{1}$ the difference between the means of the arms on treatment A, and $d_{2}$ difference between means of the arms on treatment B.

In all the power simulations and computations, proportion of participants choosing treatment A vs B in the choice arm was set to 40%, and sample size was fixed to 400. In the case of Normally distributed outcomes, the parameters $\left(\beta_{C0},\beta_{CT},\beta_{R0},\beta_{RT}\right)$, where $\beta_{CT}$ and $\beta_{RT}$ defined as the change in the mean outcome in treatment A relative to treatment B, were set as follows: $\beta_{C0}=\beta_{R0}=6$, $\beta_{RT}=0$, and $\beta_{CT}$ was varied between −4 to 4 in increments of 1. In the case of Poisson using log link, parameters were set to $\beta_{C0}=\beta_{R0}=1.8$ and $\beta_{RT}=0$, and $\beta_{CT}$ was set equal to log(x) with x varying between 0.1 and 1.9 in increments of 0.1. Finally, in case of Bernoulli the parameters were set to $\beta_{C0}=\beta_{R0}=0.3$ and $\beta_{RT}=0$, and $\beta_{CT}$ was set equal to log(x) with x varying between 0.1 and 1.9 in increments of 0.1.

**Fig. 3:**
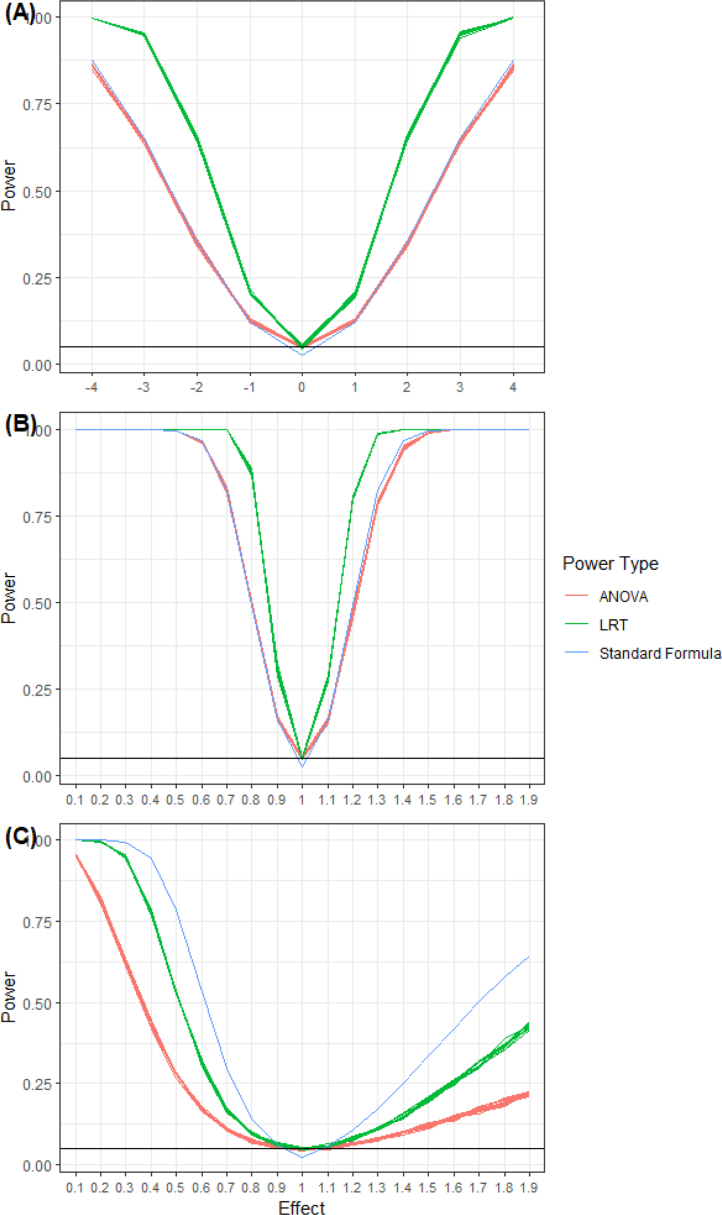
Simulated power for preference effect comparing LRT method to ANOVA and standard formula for N=400 and α=0.05. A, Normal outcomes; B, Poisson outcome; C, Bernoulli outcome.

As can be seen from Fig. 3 where outcomes follow Normal and Poisson distributions, LRT consistently has higher power values relative to ANOVA and the standard sample size formula, giving it an advantage of requiring a smaller sample for the same power and effect size. In case of Bernoulli outcomes, LRT has higher simulated power than ANOVA again making it more efficient to use when designing and analyzing data from these type of studies. On the other hand, calculated power based on the standard formula is much higher than ANOVA, simulated power version of the standard formula, and LRT simulated power, which implies that using this standard sample size formula is not appropriate for these designs when the outcome is binary. If one uses the standard formula to determine the sample size and uses ANOVA or LRT for analysis this may result in an underpowered study.

## 4 Application to women take PRIDE study

Women Take PRIDE (WTP) intervention study [10] aimed to compare modes of deliverance, Group and Self-Directed, of a behavioral intervention program for Women 60 years or older with heart disease being treated with daily heart medication. The intervention program introduced participants to a process of identifying and resolving problems they encounter in managing their heart disease. PRIDE was chosen as an acronym for the self-regulatory steps that program participants were asked to follow: problem selecting, researching one’s daily routine, identifying a behavioral goal, developing a plan to reach one’s goal, and establishing reward for making progress [17]. The intervention program for the Group format consisted of weekly 2.5-h meetings of six to eight women for six weeks. The Self-Directed group were provided with the same content as the Group program but were allowed to complete it at their own pace. Participants were followed at four, twelve, and eighteen months by telephone interviews and clinical assessment.

The WTP study used a hybrid clinical trial design such as the one described in this paper. Potential participants were randomized between choice and random groups before they were contacted, and they were not informed of the existence of the other group. Next, participants in the random group were randomized again into three groups: Group program, Self-Directed program, and control. Individuals in the control group were assigned the standard care. Participants in the choice group were given their choice of treatment, Group or Self-Directed. Janevic et al. [8] reported a total of 1071 participants in the study with 575 in the random group, and 496 in the choice group. For our paper, we were only able to acquire a subset of the data that excluded information about the control arm and only had 391 participants in the random group and 480 in the choice group.

The 6-min walking distance (6MWD) is an inexpensive and simple test that measures the ability to walk for a maximum of 6 min. In this study, 6MWD was measured as a continuous outcome in feet. According to [10], 6MWD is an objective measure of functional status. Given that heart problems may impair patient’s physical activity, we illustrate our proposed method by choosing change in 6MWD for our outcome to assess the effect of the treatments on the change between baseline and 4 months.

From 871 participants, 172 had missing 6MWD at baseline (84 and 88 in random and choice groups, respectively) and 381 at 4 months follow-up (171 and 210 in the random and choice groups, respectively). Thus, the final sample size for this illustration was 443. We did not attempt to use imputation methods to address missingness as this is not the focus of this illustration. Of the 198 participants randomized to the random group, 102 (52%) in the random group were randomized to Self-Directed program. On the other hand, 85 (35%) of 245 randomized to the choice group chose the Self-Directed program (see Table S.4 for descriptive summary statistic of the outcome of interest). Looking at the demographics and health characteristics, we did not find any significant differences between the choice and random groups (Table S.3). However, we decided to use SIP physical score as a covariate as it is used to evaluate the effect of disease on physical functioning [18]. We decided on following the analysis plan summarized in Fig. 1.

For our illustration, we dichotomized the outcome into improve vs. not improve if the change in 6MWD is positive vs. negative or zero, respectively. Next, we fitted the proposed model ((3) & (4)) to estimate six parameters (random intercept, choice intercept, random self directed, choice self-directed, effect of SIP, and preference effect). We observed that the Self-Directed format had a higher estimated odds of improving than Group format group for participants who got to choose their mode of deliverance, but had lower estimated odds for participants randomized to that treatment (Table 3).

Using our proposed LRT method to test for the preference effect, we did not get a significant result (p = 0.5935). Consequently, we assumed that the treatment effect is similar for both the choice and random groups. We proceeded to fit a single model combining data from both random and choice groups to estimate the treatment effect. We found that the treatment effect was not significant (estimate = 0.02 with p = 0.9410) implying that we found no evidence to show that there is a difference in the improvement in the 6MWD between the different format groups.

As a sensitivity analysis, we analyzed the study using proposed LRT without covariate, using ANOVA approach, and using a naive approach that uses a traditional logistic model with the group indicator as predictor in the model. Results from all approaches were non-significant. We were unable to directly compare between ANOVA and proposed LRT because of the way the effects in the ANOVA model are defined ([2]). Results are available in the supplementary materials Tables S.5-S.7. We would like to note that the proposed LRT approach showed that SIP physical score is negatively associated with 6MWD improvement (p-value = 0.0410), which is expected as higher SIP physical scores represent greater physical dysfunction status. This implies that SIP may be a moderator for 6MWD and should be included in the model, which we are not able to do with the ANOVA approach.

Furthermore, we conducted a post-hoc power analysis to determine the required number of participants for detecting a preference effect. Assuming a 50-50 allocation between random and choice groups, and utilizing the observed percentage of participants selecting the Self-Directed program (35%) along with the estimates from Table 3, we determined that a minimum of 2500 participants is needed to detect a significant preference effect.

**Table 3:** Parameter estimates of model for Women Take PRIDE Study.

Parameter	Estimate	p-value
Random intercept	0.4395	0.1489
Random self directed	−0.1240	0.7873
Choice intercept	0.1211	0.6590
Choice self directed	0.0821	0.8548
Cov SIP at 4-month follow-up	−0.0322	0.0410
Preference effect		0.5935

## 5 Discussion

The number of studies incorporating participant’s preference in clinical trials is growing, especially for designs based on health interventions like exercise programs and health education. New methods that allow for the inclusion of covariates in these type of designs are needed.

In this paper, we propose an alternative to the ANOVA methods that accommodates covariates in the model. Based on our research, we did not find any publish work akin to ANCOVA for testing the preference effect for trials incorporating participant’s choice of treatment. This gives our proposed method an advantage over the ANOVA if investigators want to include covariates in their analysis. In the absence of covariates, the performance of the proposed method compared to ANOVA with regard to the simulated type I error rates were similar in the cases of Normal and Poisson. In the case of Bernoulli where the sample size is small (n = 100), our method had inflated type I simulated error rates. However, n = 100 for this type of design where participants are assigned to 4 groups is not recommended. Instead, we recommend at least a sample size of 400 especially when the outcome is binary with probability of an event far from 0.5. In this case, the proposed method performed well relative to both the target type I error rate and the simulated type I error rate for ANOVA.

Looking at power analysis, we noticed that simulated LRT performed slightly better than ANOVA, giving it the advantage of reaching the sought after power using a smaller sample while allowing for the inclusion of covariates in the analysis. Similar results were shown by Chahine and Aban [19], when examining survival data using the Weibull distribution. We believe this is due to LRT using the sufficient statistic, i.e., no loss of information about the parameters of interest. ANOVA is based on sample means which may not necessarily be sufficient statistics. An example of this difference in power when designing a study with normal outcome for an effect of 3, a sample of 400 allows the study to reach 90% power using our LRT approach while only 63% power using the ANOVA approach. Power simulations were illustrated by choosing ϕ, the proportion of participants choosing one treatment vs another to be 40%. If ϕ deviate farther from 50% (near 0 or near 1), a larger sample will be needed for large-sample approximation to perform well as smaller number of participants will choose (or not choose) the treatment.

It is worth noting that using the standard formula for sample size computations when outcome is binary, although convenient relative to simulations, may lead to an underpowered study. For binary type outcome, we recommend investigators to consider using either simulated LRT or ANOVA power to determine the sample size. We also recommend to run a pilot study surveying patients about their treatment choice before proposing a trial design as this information will be necessary in sample size computations.

Finally, we provided an illustration of the proposed method using a subset of the Women Take PRIDE study data using a binary outcome. We showed the suggested steps needed in analyzing the data to help guide researchers in how to implement the method.

## 6 Limitations and future work

The goal of this work is to propose an alternative approach to analyze hybrid trials incorporating patient’s treatment choice. Comparing our work to [7], we have the disadvantage of not allowing for inclusion of covariates affecting the proportion of participants choosing one treatment versus another. In the future, we plan to extend our work to incorporate covariates that may only affect the participant’s choice in the analysis and compare it to the EM method suggested by Long et al.

In this paper, we assumed that all participants in the choice arm have a treatment preference, it is possible that some participants may be unable to make a choice. These undecided participants form a particular group that need to be considered. Walter et al. [20] suggested methods to address this. It will be useful to extend the suggested methodology to account for this possibility.

A caveat of using likelihood approach is the sensitivity of the method to distribution misspecification. We recommend following the common practice of checking the distribution of the observed data before applying the proposed method.

## CRediT authorship contribution statement

**Rouba Chahine:** Writing – review & editing, Writing – original draft, Software, Methodology, Formal analysis, Conceptualization. **Inmaculada Aban:** Writing – review & editing, Methodology, Conceptualization.

## Declaration of competing interest

The authors declare that they have no known competing financial interests or personal relationships that could have appeared to influence the work reported in this paper.

## Data Availability

The authors do not have permission to share data.

## Appendix A Supplementary materials

Additional simulation results supporting Section 3.1 are available in Appendix A. In Appendix B, we provide the baseline characteristics table for the Women Take Pride study, along with descriptive summary statistics of the outcomes and additional modeling results tables. Sample R code can be found in Appendix C.

MMC S1 .
