# Supplementary material for: Likelihood based inferences for trials incorporating participant’s treatment choice
Source: Contemp Clin Trials Commun. 2024 May 15;39:101306. doi: 10.1016/j.conctc.2024.101306 (PMC11170208; doi:10.1016/j.conctc.2024.101306)
Supplement: MMC S1 — . [file mmc1.pdf]

Supporting Information for Likelihood Based Inferences for Trials Incorporating Patient's Treatment Choice by Rouba Chahine and Inmaculada Aban

Appendix A

In this section, we provide extra tables for evaluating the LRT using different scenarios.

Table S. 1: Summary of the simulated distribution of the MLEs based on 4000 replications of Normal data with covariates

	Case 1: Continuous Covariate		Case 2: Binary Covariate	
	True value	Estimates (Std)	True value	Estimates (Std)
β_{R0}	12	12.015 (0.861)	12	12.0 (0.561)
β_{RT}	3	2.994 (0.702)	3	2.995 (0.691)
β_{C0}	13	13.0 (0.945)	13	13.005 (0.538)
β_{CT}	3	3.021 (0.781)	3	3.007 (0.742)
α	-0.1	-0.10 (0.018)	2	1.999 (0.502)

Table S. 2: Summary of the simulated distribution of the MLEs based on 4000 replications of Bernoulli data using different proportions

	Case 1 ($p \approx 0.25$)		Case 2 ($p \approx 0.50$)		Case 3 ($p \approx 0.75$)	
	True value	Bias (Std)	True value	Bias (Std)	True value	Bias (Std)
β_{R0}	-1	-0.016 (0.235)	0.2	0.004 (0.203)	1	0.016 (0.235)
β_{RT}	0	0.009 (0.324)	0	-0.005 (0.293)	0	-0.010 (0.325)
β_{C0}	-1	-0.012 (0.207)	0.2	0.003 (0.182)	1	0.012 (0.207)
β_{CT}	0.3	0.003 (0.319)	0.3	0.005 (0.299)	0.3	0.013 (0.353)

Appendix B

In this section, we provide tables for the Application to Women Take PRIDE Study.

Table S. 3: Baseline demographic and health characteristics

	Total sample (n=443)	Random Group (n=198)	Choice Group (n=245)	p-value
Demographics				
Age, mean (range)	73 (60-87)	73 (61-87)	73 (60-87)	0.4146
Race, n (\% white)	389 (88)	169 (86)	220 (90)	0.1972
Education, n (\%)			0.1416	
Less than high school	65 (15)	32 (16)	33 (13)	
High school	177 (40)	69 (35)	108 (44)	
At least some college	201 (45)	97 (49)	104 (42)	
Employed, n (\%)	45 (10)	17 (9)	28 (11)	0.3248
Income, n (\%)				0.2467
More than $20K	291 (69)	136 (72)	155 (67)	
Married, n (\%)	232 (52)	109 (55)	123 (50)	0.3099
Living alone, n (\%)	156 (36)	62 (32)	94 (39)	0.1432
Social support (0-5), median (IQR)	1.5 (1-2)	1.5 (1-2)	1.5 (1-2)	0.2321
Health characteristics				
Number of comorbidities, median (IQR)	1 (0-2)	1 (0-2)	1 (1-2)	0.1745
Cardiac severity (0-70) , median (IQR)	3 (0-9)	3 (0-9)	4 (0-10)	0.1934
SIP physical score (0-100), mean (SD)	7.42 (8.58)	7.19 (8.77)	7.60 (8.44)	0.1432
Depression score (0-24), median (IQR)	1 (0-4)	1 (0-4)	1 (0-5)	0.2642
6-minute-walk in feet, mean (SD)	785.19 (449.40)	786.87 (456.35)	783.83 (444.63)	0.9643

Table S. 4: Descriptive summary statistic of the outcomes

	Total sample (n=445)	Random Group (n=198)	Choice Group (n=245)
Baseline 6MWD			
Mean (STD)	785.19 (449.40)	786.87 (456.35)	783.83 (444.63)
Range	(11.92 , 1820)	(50 , 1820)	(11.92 , 1555)
Median (IQ)	800 (320 , 1200)	800 (310 , 1200)	732 (380 , 1198)
4 Month 6MWD			
Mean (STD)	804.77 (463.41)	822.57 (470.29)	790.39 (458.23)
Range	(50 , 1840)	(50 , 1840)	(50 , 1660)
Median (IQ)	810 (385 , 1240)	900 (380 , 1235)	800 (385 , 1250)
Improve			
N(%)	226 (51)	107 (54)	119 (49)

Table S. 5: Results using logistic model accounting for group difference (Choice vs Random) and SIP as covariates

Parameter	Estimate	p-value
Intercept	0.122	0.489
Treatment Effect	0.080	0.770
Group Effect	0.318	0.225
Preference Effect (interaction treatment*Group)	-0.204	0.607
SIP	-0.032	0.008

Table S. 6: Results using ANOVA approach without covariates

Effect	Statistic	p-value
Treatment	0.117	0.453
Selection (Choice)	-0.734	0.232
Preference	-1.204	0.114

Table S. 7: Results using proposed LRT approach without covariates

Parameter	Estimate	p-value
Random interncept	0.2513	0.4145
Random Self Directed	-0.1728	0.7011
Choice intercept	-0.0750	0.7352
Choice Self Directed	0.0515	0.9049
Prefernce effect		0.5764

Appendix C

In this section, we provide sample R codes that were used for the application

```
library(tidyverse)

#get the data
data<-as_tibble()

#rename data varaibles to match likelihood variables
dat<-data %>% #SELFD=Treatment A
mutate(T=SELFD, R=1-CHOICE, y=improve, x=SIP4) %>% #/BLV2027
drop_na()
#**************************************************************************
#Full Model
#**************************************************************************
loglik_full <- function(param, y, x, T, R){
#the parameter to be estimated
betaR0<-param[1]
betaRT<-param[2]
betaC0<-param[3]
betaCT<-param[4]
alpha<-param[5]
#Expected value of y
temp<- (R*(betaR0 + betaRT*T+alpha*x) + (1-R)*(betaC0 + betaCT*T+alpha*x))
mu<-exp(temp)/(1+exp(temp))
#loglik
perpat <- y*log(mu)+(1-y)*log(1-mu)
#sum of the logs
negloglik <- -sum(perpat, na.rm=TRUE)
#add this code to avoid log(0)
if ((is.na(negloglik)) | (is.nan(negloglik))) {negloglik <- 1E10}
if (negloglik==Inf) {negloglik <- 1E10}
if (negloglik>1E10) {negloglik <- 1E10}
return(negloglik)
}
#**************************************************************************
#Test cov effect
```

```
#***************************************************************************
loglik_alpha <- function(param, y, x, T, R){
#the parameter to be estimated
betaR0<-param[1]
betaRT<-param[2]
betaC0<-param[3]
betaCT<-param[4]
#Expected value of y
temp<- (R*(betaR0 + betaRT*T) + (1-R)*(betaC0 + betaCT*T))
mu<-exp(temp)/(1+exp(temp))
#loglik
perpat <- y*log(mu)+(1-y)*log(1-mu)
#sum of the logs
negloglik <- -sum(perpat, na.rm=TRUE)
#add this code to avoid log(0)
if ((is.na(negloglik)) | (is.nan(negloglik))) {negloglik <- 1E10}
if (negloglik==Inf) {negloglik <- 1E10}
if (negloglik>1E10) {negloglik <- 1E10}
return(negloglik)
}
#***************************************************************************
#Test for intercept in random group
#***************************************************************************
loglik_R0 <- function(param, y, x, T, R){
#the parameter to be estimated
betaRT<-param[1]
betaC0<-param[2]
betaCT<-param[3]
alpha <- param[4]
#Expected value of y
temp<- (R*(betaRT*T+alpha*x) + (1-R)*(betaC0 + betaCT*T+alpha*x))
mu<-exp(temp)/(1+exp(temp))
#loglik
perpat <- y*log(mu)+(1-y)*log(1-mu)
#sum of the logs
negloglik <- -sum(perpat, na.rm=TRUE)
#add this code to avoid log(0)
if ((is.na(negloglik)) | (is.nan(negloglik))) {negloglik <- 1E10}
if (negloglik==Inf) {negloglik <- 1E10}
if (negloglik>1E10) {negloglik <- 1E10}
return(negloglik)
}
#***************************************************************************
#Test for treatment effect in random group
#***************************************************************************
loglik_RT <- function(param, y, x, T, R){
#the parameter to be estimated
```

```
betaR0<-param[1]
betaC0<-param[2]
betaCT<-param[3]
alpha<-param[4]
#Expected value of y
temp<- (R*(betaR0 + alpha*x) + (1-R)*(betaC0 + betaCT*T+alpha*x))
mu<-exp(temp)/(1+exp(temp))
#loglik
perpat <- y*log(mu)+(1-y)*log(1-mu)
#sum of the logs
negloglik <- -sum(perpat, na.rm=TRUE)
#add this code to avoid log(0)
if ((is.na(negloglik)) | (is.nan(negloglik))) {negloglik <- 1E10}
if (negloglik==Inf) {negloglik <- 1E10}
if (negloglik>1E10) {negloglik <- 1E10}
return(negloglik)
}
#****************************************************************************
#Test for intercept in Choice group
#****************************************************************************
loglik_C0 <- function(param, y, x, T, R){
#the parameter to be estimated
betaR0<-param[1]
betaRT<-param[2]
betaCT<-param[3]
alpha<-param[4]
#Expected value of y
temp<- (R*(betaR0 + betaRT*T+alpha*x) + (1-R)*( betaCT*T+alpha*x))
mu<-exp(temp)/(1+exp(temp))
#loglik
perpat <- y*log(mu)+(1-y)*log(1-mu)
#sum of the logs
negloglik <- -sum(perpat, na.rm=TRUE)
#add this code to avoid log(0)
if ((is.na(negloglik)) | (is.nan(negloglik))) {negloglik <- 1E10}
if (negloglik==Inf) {negloglik <- 1E10}
if (negloglik>1E10) {negloglik <- 1E10}
return(negloglik)
}
#****************************************************************************
#Test for treatment effect in random group
#****************************************************************************
loglik_CT <- function(param, y, x, T, R){
#the parameter to be estimated
betaR0<-param[1]
betaRT<-param[2]
betaC0<-param[3]
```

```
alpha<-param[4]
#Expected value of y
temp<- (R*(betaR0 + betaRT*T+alpha*x) + (1-R)*(betaC0 + alpha*x))
mu<-exp(temp)/(1+exp(temp))
#loglik
perpat <- y*log(mu)+(1-y)*log(1-mu)
#sum of the logs
negloglik <- -sum(perpat, na.rm=TRUE)
#add this code to avoid log(0)
if ((is.na(negloglik)) | (is.nan(negloglik))) {negloglik <- 1E10}
if (negloglik==Inf) {negloglik <- 1E10}
if (negloglik>1E10) {negloglik <- 1E10}
return(negloglik)
}
#***************************************************************************
#Test for preference effect
#***************************************************************************
loglik_null <- function(param, y, x, T, R){
#the parameter to be estimated
beta0<-param[1]
betaT<-param[2]
alpha <- param[3]
#Expected value of y
temp<-(beta0 + betaT*T +alpha*x)
mu<-exp(temp)/(1+exp(temp))
#loglik
perpat <- y*log(mu)+(1-y)*log(1-mu)
#sum of the logs
negloglik <- -sum(perpat, na.rm=TRUE)
#add this code to avoid log(0)
if ((is.na(negloglik)) | (is.nan(negloglik))) {negloglik <- 1E10}
if (negloglik==Inf) {negloglik <- 1E10}
if (negloglik>1E10) {negloglik <- 1E10}
return(negloglik)
}
#***************************************************************************
# Perform the tests
#***************************************************************************
# Get needed info from data
choice<-dat%>%
filter(R==0)
N<-length(dat$y)
m<-length(choice)
m1<-sum(choice$T==1)
phi=m1/m
#### LRT ####################################################################
#Full
```

```
fun_full = optim(par=c(0.01, 0.01, 0.01, 0.01, 0.01), fn=loglik_full, y=dat$y
temp_full <- (dat$R*(fun_full$par[1] + fun_full$par[2]*dat$T+fun_full$par[5]*
mu_full<-p(temp_full)
perpat_full<-f(mu=mu_full,  y=dat$y)
log_L_full=sum(log(perpat_full))

#Null
fun_null = optim(par=c(0.01, 0.01, 0.01), fn=loglik_null, y=dat$y, x=dat$x, T
temp_null <-(fun_null$par[1]+fun_null$par[2]*dat$T+fun_null$par[3]*dat$x)
mu_null<-p(temp_null)
perpat_null <-f(mu=mu_null, y=dat$y)
log_L_null <- sum(log(perpat_null))

#Cov significance
fun_alpha=optim(par=c(0.01, 0.01, 0.01, 0.01), fn=loglik_alpha, y=dat$y, x=da
temp_alpha <- (dat$R*(fun_alpha$par[1] + fun_alpha$par[2]*dat$T) + (1-dat$R)*
mu_alpha<-p(temp_alpha)
perpat_alpha<-f(mu=mu_alpha,  y=dat$y)
log_L_alpha=sum(log(perpat_alpha))

#Beta R0
fun_R0 = optim(par=c(0.01, 0.01, 0.01, 0.01), fn=loglik_R0, y=dat$y, x=dat$x,
temp_R0 <- (dat$R*( fun_R0$par[1]*dat$T+fun_R0$par[4]*dat$x) + (1-dat$R)*(fun
mu_R0<-p(temp_R0)
perpat_R0<-f(mu=mu_R0,  y=dat$y)
log_L_R0=sum(log(perpat_R0))

#Beta RT
fun_RT = optim(par=c(0.01, 0.01, 0.01, 0.01), fn=loglik_RT, y=dat$y, x=dat$x,
temp_RT <- (dat$R*(fun_RT$par[1] +fun_RT$par[4]*dat$x) + (1-dat$R)*(fun_RT$pa
mu_RT<-p(temp_RT)
perpat_RT<-f(mu=mu_RT,  y=dat$y)
log_L_RT=sum(log(perpat_RT))

#Beta C0
fun_C0 = optim(par=c(0.01, 0.01, 0.01, 0.01), fn=loglik_C0, y=dat$y, x=dat$x,
temp_C0 <- (dat$R*(fun_C0$par[1] + fun_C0$par[2]*dat$T+fun_C0$par[4]*dat$x) +
mu_C0<-p(temp_C0)
perpat_C0<-f(mu=mu_C0,  y=dat$y)
log_L_C0=sum(log(perpat_C0))

#Beta CT
fun_CT = optim(par=c(0.01, 0.01, 0.01, 0.01), fn=loglik_CT, y=dat$y, x=dat$x,
temp_CT <- (dat$R*(fun_CT$par[1] + fun_CT$par[2]*dat$T+fun_CT$par[4]*dat$x) +
mu_CT<-p(temp_CT)
perpat_CT<-f(mu=mu_CT,  y=dat$y)
log_L_CT=sum(log(perpat_CT))
```

```
#### The tests!!!!!##########################################################

#Likelihood Ratio Test
LRT<- -2*(log_L_null - log_L_full)
LRT_P_Value<-pchisq(LRT, 1-error, df=2, lower.tail=F)

alpha_LRT <-  -2*(log_L_alpha - log_L_full)
alpha_P_Value<-pchisq(alpha_LRT, 1-error, df=1, lower.tail=F)

R0_LRT <-  -2*(log_L_R0 - log_L_full)
R0_P_Value<-pchisq(R0_LRT, 1-error, df=1, lower.tail=F)

RT_LRT <-  -2*(log_L_RT - log_L_full)
RT_P_Value<-pchisq(RT_LRT, 1-error, df=1, lower.tail=F)

C0_LRT <-  -2*(log_L_C0 - log_L_full)
C0_P_Value<-pchisq(C0_LRT, 1-error, df=1, lower.tail=F)

CT_LRT <-  -2*(log_L_CT - log_L_full)
CT_P_Value<-pchisq(CT_LRT, 1-error, df=1, lower.tail=F)
```
